# Okra (*Abelmoschus esculentus* L.) Flour Integration in Wheat-Based Sourdough: Effect on Nutritional and Technological Quality of Bread

**DOI:** 10.3390/foods13203238

**Published:** 2024-10-11

**Authors:** Francesca Valerio, Mariaelena Di Biase, Valentina Cifarelli, Stella Lisa Lonigro, Amina Maalej, Stella Plazzotta, Lara Manzocco, Sonia Calligaris, Hana Maalej

**Affiliations:** 1 Institute of Sciences of Food Production, National Research Council, Via G. Amendola 122/O, 70126 Bari, Italy; mariaelena.dibiase@ispa.cnr.it (M.D.B.); valentina.cifarelli@ispa.cnr.it (V.C.); lisa.lonigro@ispa.cnr.it (S.L.L.); sonia.calligaris@uniud.it (S.C.); 2 Laboratory of Environmental Bioprocesses, Centre of Biotechnology of Sfax, Sfax 3018, Tunisia; maalejamina@yahoo.fr; 3 Department of Agricultural, Food, Environmental and Animal Sciences, University of Udine, via Sondrio 2/A, 33100 Udine, Italy; stella.plazzotta@uniud.it (S.P.); lara.manzocco@uniud.it (L.M.); 4 Laboratory of Biodiversity and Valorization of Arid Areas Bioresources (BVBAA), LR16ES36, Faculty of Sciences of Gabes, University of Gabes, Gabes 6072, Tunisia; hana.maalej@univgb.tn

**Keywords:** lactic acid bacteria, fermentation, protein content, amino acids, organic acids, l-glutamic acid, polysaccharides, wheat bread

## Abstract

The aim of this study was to develop an innovative sourdough using dehydrated okra (*Abelmoschus esculentus* L.) pod flour and to use it in the production of bread. Three different flours (sun-dried S, freeze-dried F, oven-dried O) were individually mixed at 9% with wheat flour (Dough Yield 300) and fermented (N_0_: 8.0 log_10_ CFU/g) for 14 h, using *Lactiplantibacillus plantarum* ITM21B, *Weissella cibaria* C43-11 or *Leuconostoc mesenteroides* C43-2M. The results showed that after fermentation, the content of organic acids (lactic, acetic and propionic), exopolysaccharides (EPS), l-glutamic acid and total free amino acids (TFAA) increased and the high molecular weight proteins were converted into smaller proteins. Sourdough based on *Leuc. mesenteroides* and O flour (O_LeuMes) was selected to evaluate its applicability in bread making. It was included in the yeast-leavened bread formulation at 20 or 40% (0.6% and 1.21% *w/w* O flour replacement). The results showed that fermentation limited the negative effects of unfermented O flour on bread quality attributes, mainly the specific volume and firmness. Bread with O_LeuMes at 40% was improved in TFAA, EPS and l-glutamic acid content and showed a higher specific volume and lower moisture and firmness compared to bread with the unfermented O flour.

## 1. Introduction

Okra (*Abelmoschus esculentus* L.), commonly known as gumbo or lady’s finger, belongs to the *Malvaceae* family and is an easily available, renewable and inexpensive natural source. It is consumed in the Mediterranean region and can be considered as a functional food since it is rich in minerals, protein, fat, phenolic compounds and polysaccharides [1,2]. In Tunisia, okra is cultivated in Djérid, a semi-desert region in the south-west of the country, in the areas of Sidi Daoud near La Marsa, and in the north-west of the country in Béja, Jendouba and Bizerte [3]. Given the growing interest in the Mediterranean diet, this plant could be valorized as a healthy ingredient applicable in many food products, especially in cereal-based foods, which represent up to 77% of the total caloric consumption in Africa [2,4,5]. Okra is rich in soluble dietary fiber in the form of gums and pectins, which help to lower serum cholesterol levels, thereby reducing the risk of heart disease [6]. The health-promoting capacity of okra is mainly related to its polysaccharides [7,8], which are typical pectic polysaccharides containing abundant rhamnogalacturonan I (RG I) with repeating disaccharide units → 4)-d-GalpAMe-(1 → 2)-l-Rhap-(1 → as backbone and neutral sugars of galactose and arabinose as side chains [9,10]. Okra polysaccharides possess significant bioactivities such as anti-fatigue [11], antioxidant [12], anti-hyperlipidemic [2] and anti-hyperglycemic effects [7] as well as interesting potential as prebiotics [13]. In addition, the polysaccharides in the seedless pod of okra form a slimy viscous system in water [14]. Due to its high moisture content, fresh okra is known to be highly perishable at room temperature, resulting in deterioration of its chemical, physical and biological properties. A dehydration process would therefore be required to allow its safe use as a food ingredient and to preserve its bioactive compounds. However, it should be emphasized that the technology used to remove moisture (e.g., sun drying, oven drying, freeze drying) could deeply affect the health and technological functionalities of dried plant flour [15].

Innovation in the bakery sector is currently exploring biotechnological processes, including the combination of alternative flours as sources of carbohydrates, proteins, vitamins and minerals to wheat flour, in order to improve the nutritional, functional and technological qualities of products [16]. White wheat bread has a high glycemic index (GI), i.e., it rapidly raises the postprandial blood glucose levels. For this reason, the bio-functional properties of okra fruits may be particularly relevant to enriching the nutritional properties of wheat bread by reducing its starch digestion rate and extension. Soluble dietary fibers, such as okra pod mucilage, could form viscous solutions or gels, affecting passage rate, viscosity and interactions with digestive enzymes and bile salts in the stomach and small intestine [17]. Recently, some studies have reported the potential of okra flour or derived hydrocolloids in the production of bread, with the ultimate aim of enriching bread with prebiotic polysaccharides useful for intestinal microflora, phenolic compounds, and micro- and macro-nutrients [18,19,20,21]. Other health benefits of okra consumption are also related to its phenolic content, since such compounds have beneficial biological activities, including the ability to modulate oxidative and inflammatory stress [17]. However, fortification of bread with ingredients containing phenolic compounds could slow down starch hydrolysis through the potential inhibition of digestive enzymes (i.e., α-amylase and glucosidases) [22] and glucose transporters in the intestinal brush border [23,24,25]. The application of okra derivatives in bread making could reveal other technological drawbacks, mainly related to the soluble fiber content of the polysaccharidic fraction, which retains water, thus negatively affecting wheat-gluten development during bread manufacturing [26]. As has been widely demonstrated, the textural and sensorial qualities of fiber-enriched breads can be significantly improved by sourdough fermentation, even in the presence of hydrocolloids such as pectin [27]. The sourdough process is a well-known ancient and sustainable technology characterized by the simultaneous presence of lactic acid bacteria (LAB) and yeasts in a dough which determines the transformation of food raw materials. During the process, several compounds are produced such as hydrocolloids, organic acids and enzymes, flavor and taste-active compounds and antimicrobial substances, which have technological, functional and health properties. The process can occur spontaneously or can be activated by specific strains [28]. Among the commonly used microorganisms applied in sourdough, LAB contribute to improving the quality, shelf life and functional properties of fermented foods by converting food components into added-value compounds [29]. They also contribute to modifying the bioaccessibility and bioavailability of polyphenols mainly through the release of free phenolic acids by the esterase activity of sourdough microorganisms [30,31]. Therefore, the combination of sourdough fermentation and the incorporation of a rich source of phenolics such as okra flour could provide an additional functional trait to the resulting bread. At the same time, the addition of a rich source of phenolics to food formulation should consider the associated safety aspects in order to select the optimal percentage of incorporation. Altunkaya et al. [32] evaluated the chemical safety of wheat bread fortified with pomegranate peel powder using the *Artemia salina* assay. The authors observed an increase in toxicity as a function of the replacement of wheat flour with the plant by-product, thus suggesting that the final products should be tested for safety aspects. 

During fermentation, LAB activate many metabolic activities, leading to the production of antimicrobials and exopolysaccharides (EPS), acidification and redox potential modification, oligosaccharides and glutamine and glutamate metabolism [33]. The EPS, divided into homopolysaccharides (HoPS) and heteropolysaccharides (HePS), can be used instead of hydrocolloids as texturizing, antistaling or prebiotic additives in bread, improving its textural and nutritional characteristics [34]. 

To date, okra has mainly been subjected to spontaneous fermentation, resulting in the improvement in nutrient composition, antioxidants and some functional properties [35,36]. Recently, Wang et al. [37] used okra juice as a fermentation substrate using a *Lactiplantibacillus plantarum* strain to evaluate the effect of the process on the physico-chemical properties, antioxidant activity and immunomodulatory ability of polysaccharides. Until now no data have been reported on the fermentation of okra flours in a wheat-based sourdough formulation by using selected starter strains. 

Based on the above considerations, the aim of this study was to evaluate the suitability of dehydrated okra pods as functional food ingredients after fermentation. Specifically, okra flours produced by three different drying technologies (sun drying, air drying, freeze drying) were fermented in combination with wheat flour to produce sourdoughs, which were further used for bread preparation. The results are the starting point for expanding the application of okra in food products, allowing its valorization.

## 2. Materials and Methods

The list of abbreviations used in this research can be found in the Abbreviations Section.

### 2.1. Raw Materials 

The ingredients used in this study were commercial soft wheat flour type 0 (fat 2%, carbohydrates 72%, fiber 2%, protein 11%) (Molino Casillo S.p.A, Corato, Italy) and 3 types of okra flour: sun-dried okra flour (S, direct exposure to solar radiation at ≃30 °C for 24 h), oven-dried okra flour (O, drying in a forced air oven for 48–72 h) and freeze-dried okra flour (F, freezing at −80 °C overnight and followed by freeze drying for 72–96 h). All okra flour samples with particle size less than 300 μm were provided by the University of Gabes, Tunisia. The chemical composition of the okra flours obtained is reported in Table 1.

### 2.2. Microorganisms

For the production of sourdough, three LAB strains belonging to the Culture Collection of the Institute of Sciences of Food Production, National Research Council of Italy, were used as fermentation starters. In particular, the following strains were used: the proteolytic strain *Lactiplantibacillus plantarum* ITM21B, isolated from sourdough [38] and used for the production of bread with reduced salt content [39,40], *Leuconostoc mesenteroides* C43-2M and the dextran producer *Weissella cibaria* C43-11 [41], both isolated from wheat semolina [42], the latter used for the production of focaccia bread with reduced fat content [43,44]. The *W. cibaria* species, although not yet included in the Qualified Presumption of Safety (QPS) list [45] and recognized as a potential bacterial starter in food fermentation, was included in the study because it is a well characterized dextran-producer strain [41].

For long-term storage, stock cultures were prepared by mixing 800 μL of a culture in de Man Rogosa Sharpe (MRS) broth (Conda, Madrid, Spain) with 200 μL of Bacto glycerol (Fisher Scientific UK Ltd., Leicestershire, UK) and freezing this mixture at −80 °C. To obtain fresh cultures, strains were subcultured twice (1% *v*/*v*) in MRS broth for 24 h before use.

### 2.3. Microbiological Analysis of Raw Materials

A sterile Bacto-peptone (Difco, Becton Dickinson, Co., Sparks, MD, USA) solution (0.1% *w/v*) was added to 20 g of each sample up to 200 g, homogenized in a blender for 2 min, serially diluted and plated on de Man Rogosa Sharpe (MRS) (Biolife Italiana S.r.l., Milan, Italy) agar containing cycloheximide (0.1 g/L) to detect LAB. Petri dishes were then incubated aerobically at 30 °C for 48 h for colony detection. 

### 2.4. Production of Sourdough 

The strains *W. cibaria* C43-11, *Leuc. mesenteroides* C43-2M and *L. plantarum* ITM21B were selected as single starters for fermentation. The strains were cultured in MRS broth and incubated at 37 °C (*L. plantarum* ITM21B) or 30 °C (*W. cibaria* and *Leuc. mesenteroides*). In the sourdough formulation, the okra flour/wheat flour ratio and water adjustments were defined using S flour. This preliminary investigation indicated that a level of okra flour higher than 9% (*w/w*) with respect to wheat flour and a dough yield (DY) lower than 300 altered the sourdough’s consistency. Therefore, final formulations were prepared by mixing 1 g of okra flour, 10 g of wheat flour and 22 mL of water in order to select the most suitable strain and fermentation conditions for the enrichment of sourdough with bioactive molecules such as amino acids, proteins, EPS, l-glutamic acid and organic acids, and a set of experiments was prepared using O, F and S flours. Mixtures were separately inoculated at 8 log CFU/g with *W. cibaria* C43-11, *Leuc. mesenteroides* C43-2M and *L. plantarum* ITM21B as starters and incubated for 14 h at the optimal growth temperature for each. Not inoculated mixtures before fermentation were used as controls (CTR): S_CTR_T0, F_CTR_T0 and O_CTR_T0. The resulting fermentation products and controls were analyzed. The experiments were performed in duplicate. The following parameters were also registered before and after fermentation: pH, titratable acidity (TTA) and total LAB count.

### 2.5. Bread-Making Test

Among the sourdough formulations, the preliminary screening did not highly differentiate the samples. Therefore, selection of the sourdough formulation suitable for bread making was based on the type of flour and the starter strain: O flour was chosen since the oven-drying technology can be easily applied in the food sector, and the strain *Leuc. mesenteroides* C43-2M was selected as an EPS-producing strain that has not yet been deeply investigated and belongs to a species included in the QPS list. Furthermore, the sourdough formulation based on O flour and *Leuc. mesenteroides* (O_LeuMes) contained the highest EPS content. 

On the basis of these considerations, the O_LeuMes sourdough was selected for the laboratory bread-making test. Bread variants were prepared according to a standard recipe as shown in Table 2. The sourdough was added at 20% and 40% *w/w* (weight of sourdough/weight of total flour) corresponding to 0.6% and 1.2% of fermented O flour on the total flour weight, giving O_20% and O_40% breads. Unfermented O flour at 0.6% and 1.2% was used to produce control breads (CTR_20% and CTR_40%, respectively) in addition to a control bread (CTR_0%) produced with wheat flour as the standard recipe. The samples were prepared using a Princess^®^ Bread Maker Homemade Deluxe, type 152007 bread machine (Princess Household appliance BV, Breda, The Netherland). All ingredients were mixed and the dough was leavened and baked using the “Basic” program. Bread samples were cooled at room temperature for two hours, sliced and stored for physico-chemical analyses.

### 2.6. Microbiological Analysis of Sourdough 

A Sterile Bacto-peptone solution (0.1% *w/v*) was added 1:10 to the sourdough and homogenized in a stomacher for 2 min. Serial decimal dilutions of each sourdough were prepared in sterile NaCl (0.85% *w/v*) + Tween 80 (0.025%), and 100 μL aliquots of each dilution was spread on MRS plates which were incubated for 48 h at the optimal growth temperature of each strain. The total LAB count was expressed as log CFU/g. The presence of starters in each sourdough was monitored as described by Valerio et al. [40]. Bacterial DNA was extracted from each colony of overnight cultures grown in MRS broth at 30 or 37 °C as previously described [46]. Genetic identification of the strains was based on a comparison of the REP-PCR profile of each isolate with the specific pattern obtained from pure cultures of *W. cibaria* C43-11, *Leuc. mesenteroides* C43-2M and *L. plantarum* ITM21B. 

### 2.7. Physico-Chemical Analyses of Sourdough and Bread Samples 

#### 2.7.1. pH and TTA Measurement

The pH of the sourdough and bread samples was recorded using a portable pH-meter (type 110, Eutech Instruments, Singapore City, Singapore) equipped with a Double Pore D electrode (Hamilton, Bonaduz, Switzerland). TTA was measured according to AOAC Method No. 981.12 [47].

#### 2.7.2. Protein Content and Protein Profile Analysis

Total proteins were extracted from sourdoughs (before and after fermentation) and bread samples under reducing conditions as reported by Valerio et al. [44]. Protein concentration was determined using the Bradford method [48] with bovine serum albumin as standard and expressed as mg protein per gram sourdough. Total protein profiles were obtained on the 2100Bioanalyzer microchip electrophoresis system (Agilent Technologies, Waldbronn, Germany) using the Lab on-a-chip (Loac) Protein230 reagent kit (14–230 kDa) as reported in Valerio et al. [44]. According to Mw, three different areas were considered in the electropherograms: A1 (14–20 kDa), A2 (21–42 kDa) and A3 (53–63 kDa), and percentages of peaks were obtained for each Mw area. All experiments were performed in duplicate.

#### 2.7.3. Determination of Organic Acids 

Samples were prepared as described by Bavaro et al. [43]. The organic acids (lactic, acetic, propionic) were separated on an HPLC system (AKTABasic10, P-900 series pump; Amersham Biosciences AB, Uppsala, Sweden) using Rezex ROA-organic acid H^+^ (8%) (7.80 × 300 mm; Phenomenex, Torrance, CA, USA) and a three-channel UV detector (Amersham Biosciences 900) set at 210 nm. The mobile phase was 0.007 mol/L H_2_SO_4_ (Fluka, Deisenhofen, Germany) pumped through the column heated to 70 °C at a flow rate of 0.6 mL/min. The amounts of organic acids were determined by integrating calibration curves obtained from relevant standards and expressed as mmol/kg sourdough. The limit of detection (LOD) and limit of quantification (LOQ) were calculated considering a signal-to-noise ratio (S/N) of 3 and 6, respectively. LOD values of the organic acids in the extracted samples were the following for sourdough: lactic acid, 0.41 μmol/mL; acetic acid, 0.68 μmol/mL; and propionic acid 0.51 μmol/mL. The LOD values for bread samples were: lactic acid, 0.228 μmol/mL; acetic acid, 5.42 μmol/mL; and propionic acid 0.41 μmol/mL. The LOQ values corresponded to 2 × LOD. The final concentration of each organic acid in the liquid sourdough was calculated taking into account concentration and/or dilution factors and expressed as mmol/kg or μmol/kg product.

#### 2.7.4. Determination of L-Glutamic Acid 

Sourdough samples were diluted (1:5) with water, centrifuged (15,000× *g*, 20 min, 4 °C) and the supernatant filtered by centrifugation (7000× *g*, 1 h, 2 °C) through a 3000 Da cut-off micro-concentrator. Bread samples were diluted (1:10) with water, centrifuged (15,000× *g*, 20 min, 4 °C) and the supernatant freeze dried. The freeze-dried samples were resuspended in a volume of sterile water to obtain 10-fold concentrated solutions. The fractions containing molecules with a molecular weight lower than 3000 Da were analysed. The l-glutamic acid content was measured as reported by Valerio et al. [40] by spectrophotometric analysis using a commercially available l-glutamic acid test kit (K-GLUT 07/12; Megazyme International, Bray, Ireland) according to the manufacturer’s instructions and using a microtiter plate spectrophotometer BioscreenC (Oy Growth Curves AB Ltd.; Helsinki, Finland). 

#### 2.7.5. EPS Quantification 

Exopolysaccharides were extracted from sourdough and bread samples as reported by Valerio et al. [44], with slight modifications. Briefly, samples were diluted with distilled water and centrifuged (8000× *g*, 10 min). The supernatants were treated with trichloroacetic acid to a final concentration of 4% and stored at 4 °C for 2 h, to remove proteins that may interfere with EPS quantification. The precipitate was removed by centrifugation (20,000× *g*, 10 min, 4 °C) and the resulting supernatants were treated with three volumes of chilled 96–99% (*v/v*) ethanol to allow EPS precipitation. The solutions were stored overnight at 4 °C. Precipitated EPS were collected by centrifugation (8000× *g*, 20 min, 25 °C), resuspended in the original volume of distilled water, dialyzed (12–14 kDa) against distilled water for 48 h at 4 °C, lyophilized and rehydrated with distilled water to the original volume. The concentration of EPS (g/kg) was determined by the phenol-sulfuric method [49], using glucose as standard (LOD 0.078 g/L or g/kg). 

#### 2.7.6. Determination of Total Free Amino Acids 

Water/salt-soluble extracts of sourdoughs and breads were prepared according to the method originally described by Osborne [50] and modified by Weiss et al. [51] and used for the analysis of total free amino acids (TFAAs) by the cadmium–ninhydrin method as reported by Doi et al. [52].

### 2.8. Technological Analyses of Bread Samples 

To evaluate the impact of sourdough and unfermented okra flour on bread quality, moisture content, water activity (a_w_), color and firmness were assessed on the crumb and specific volume on the loaf. 

#### 2.8.1. Moisture Content

The moisture content of the crumb was measured using a gravimetric method [53]. Approximately 2 g of crumb sample were dried in a vacuum oven (1.32 kPa) at 75 °C to a constant weight (12 h). 

#### 2.8.2. Water Activity

Crumb water activity was determined using a dew-point measurement system (Aqualab 4TEV, Decagon Devices, Inc., Pullman, WA, USA) at 25 ± 1 °C. 

#### 2.8.3. Bread Crumb Firmness

Bread crumb firmness was measured by a puncture test using an Instron 4301 (Instron Ltd., High Wycombe, UK). Instrument settings and operations were performed using Automated Materials Testing System software (version 5, Series IX, Instron Ltd., High Wycombe, UK). Three slices were taken from each loaf. The central part of the slice (4.0 × 4.0 × 0.9 cm) was sampled by manual cutting with a sharp knife. A uniaxial vertical compression test was performed under ambient conditions (20 ± 2 °C, ambient humidity). Samples were penetrated using a 12.7 mm diameter cylindric probe mounted on a 100 N compression head. The crosshead speed was set at 50 mm/min. Force–distance curves were obtained and firmness was taken as the force (N) required to compress the bread crumb by 0.3 cm. Five measurements were taken at different locations on the bread slice. 

#### 2.8.4. Crumb Color Evaluation 

A tristimulus colorimeter (Chromameter-2 Reflectance, Minolta, Osaka, Japan) equipped with a CR-300 measuring head was used. Petri dishes with a diameter of 90 mm and a height of 16 mm were completely filled with the sample. The instrument was standardized against a white tile. A minimum of 5 measurements were taken for each sample. Color was expressed in CIE L*, a* and b* scale parameters. 

Total color difference (ΔE*) was calculated using Equation (1):

(1) $${\Delta E}^{*}=\sqrt{{{{(L}_{r}^{*}-L_{i}^{*})}^{2}+{(a}_{r}^{*}-a_{i}^{*})}^{2}+{{(b}_{r}^{*}-b_{i}^{*})}^{2}}$$

where $L_{r}^{*}$, $a_{r}^{*}$, $b_{r}^{*}$ are color parameters of bread without okra flour (CTR_0%) and $L_{i}^{*}$, $a_{i}^{*}$, $b_{i}^{*}$ the color parameters of breads containing okra flour. Color differences can be classified as small difference (ΔE* < 1.5), distinct (1.5 < ΔE* < 3.0) and very distinct. 

#### 2.8.5. Bread Volume

The volume (mL) of the final product was obtained by rapeseed displacement according to Approved Method 10–05 [54].

### 2.9. Statistical Analysis

Data are presented as mean values ± standard error of the mean. Data relevant to sourdough and bread samples (protein content, EPS, TFAA, organic acids, l-glutamic acid) were analyzed by one-way ANOVA followed by the Fisher’s LSD test. Moisture, a_w_, color, firmness and specific volume were similarly compared using the Tukey post hoc test. Results were considered statistically significant when the *p*-value was less than 0.05. Pearson’s correlation coefficients were estimated to establish relationships between all the data registered on the sourdough and between the technological and analytical data on the bread samples. Statistical analyses were performed using Statistica 13 software (Dell Inc., Round Rock, TX, USA, 2015). 

The data were also analyzed by principal component analysis (PCA) in order to investigate the correlation between the physico-chemical parameters of sourdough. Multivariate analysis was performed using Unscrambler (version 10.1, CAMO, Oslo, Norway). 

## 3. Results and Discussion

### 3.1. Fermentation Studies

Data from microbiological analysis highlighted the presence of preexisting contamination on the okra flours: the higher microbial load was found in O flour (6.52 ± 0.24 log CFU/g), followed by S flour (5.47 ± 0.26 log CFU/g) and F flour (4.19 ± 0.72 log CFU/g). However, the starter strains were able to dominate the natural microbiota during sourdough fermentation, reaching values ranging from 9.16 to 9.74 log CFU/g (Table 3). The highest bacterial count was registered for *L. plantarum* in F_LacPla. At the same time, the ITM21B strain reached the lowest pH values in all sourdoughs with the highest TTA values registered in O_LacPla. Acidification occurring in sourdough is one of the key factors influencing gluten protein hydrolysis by activating proteolytic enzymes, degradation of antinutritional compounds, flavor profile, bread staling, bread texture and dough rheological properties, dextran functionality, improved shelf life and other features [16]. 

**Figure 1:**
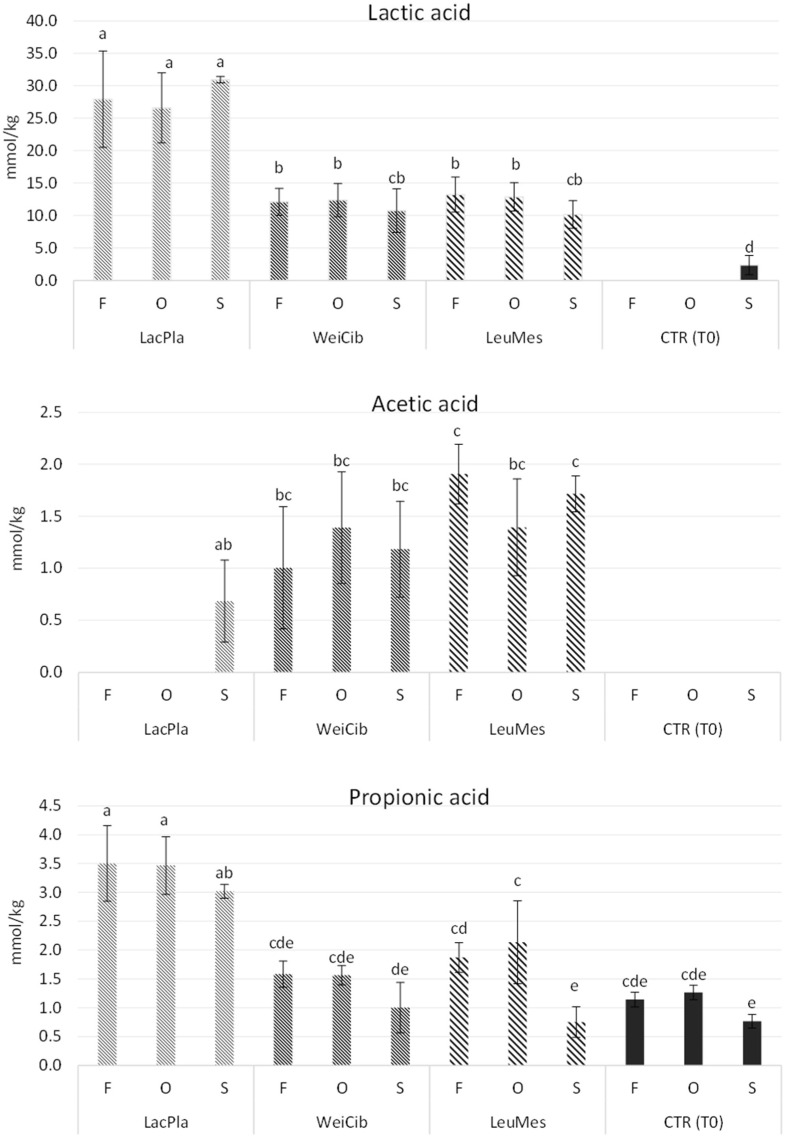
Effect of different okra flours on the content of lactic, acetic and propionic acids registered in sourdough obtained by *L. plantarum* ITM21B, *W. cibaria* C43-11 and *Leuc. mesenteroides* C43-2M after 14 h of fermentation and at start of fermentation (CTR_T0). Values with different lower-case letters are significantly different (*p* < 0.05).

All strains were able to produce the three acids monitored, with some differences in the concentrations (Figure 1). *L. plantarum* ITM21B was the highest producer of lactic acid, as already demonstrated in previous studies [44,55]. The strain *Leuc. mesenteroides* C43-2M produced acetic acid in all sourdough samples at levels significantly higher than the other strains, followed by *W. cibaria*, while strain ITM21B produced a lower amount only in S_LacPla. Propionic acid was mainly produced in ITM21B samples based on F and O flours. The production of short chain organic acids is related to the conversion of flour carbohydrates in sourdough. These compounds contribute to prolonging the bread’s shelf life, and the consequent acidification of the environment activates or inhibits many enzymatic activities [16]. Proteolytic enzymes are activated by an acidic environment, while starch-hydrolyzing enzymes are inhibited in the presence of low pH, thus resulting in slower starch digestion and a reduced glycemic response [56]. In addition, acidification enhances proteolytic activity, allowing protein breakdown and the consequent production of free amino acids involved in the Maillard reaction and the formation of volatile compounds in bread [57].

**Figure 2:**
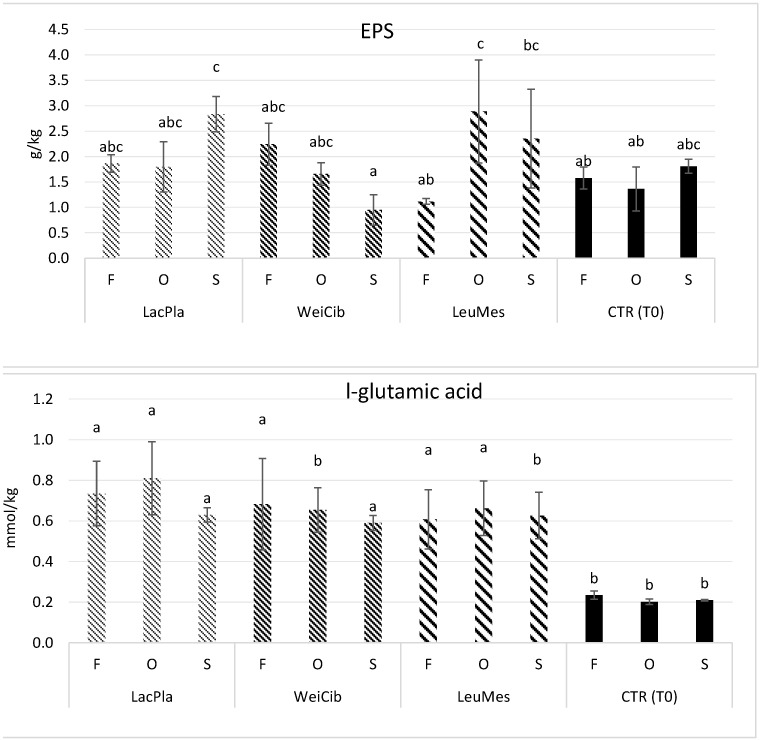
Exopolysaccharides (EPS) and l-glutamic acid content in sourdough formulations, before (CTR_T0) and after fermentation. Values with different lower-case letters are significantly different (*p* < 0.05).

Considering that okra pods contain a large amount of polysaccharides in the form of mucilage [58], which can exert technological and health activities and can be used as a carbohydrate source during fermentation, their inclusion in the form of flour in sourdough formulations can positively influence the fermentation process. At the same time, during fermentation, LAB can produce exopolysaccharides, biopolymers with technological and functional properties very similar to those of okra polysaccharides. The content of these biopolymers was registered in all the sourdough samples, although the formulation did not contain any additional carbohydrate source as a precursor of the bacterial polymers. In fact, the EPS content in each sourdough formulation was maintained at low levels although some differences were observed between samples (Figure 2). A higher level of polysaccharides was observed in O_LeuMes samples compared to the corresponding CTR. The *Leuc. mesenteroides* species is generally associated with EPS production. Recently, Bi et al. [59] prepared an HMw dextran-enriched sourdough using *Leuc. mesenteroides* ATCC 8293 as starter. The authors obtained a final product with improved bread texture and volume due to the inhibitory effect of dextran on gluten depolymerization in the acidic environment. These results confirm the positive role that polysaccharides can exert in combination with LAB fermentation.

Glutamate or l-glutamic acid, considered as a taste-active compound that can be produced during sourdough fermentation mainly in protein-rich matrices by glutamine deamidation, thus improving the taste of the relevant final product [60,61], was produced in all sourdough samples, while a lower amount (*p* < 0.05) was registered before fermentation (Figure 2). 

**Figure 3:**
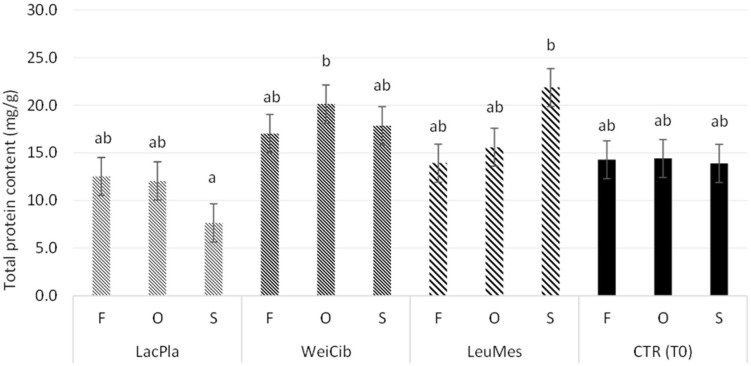
Total protein content in sourdough formulations, before (CTR_T0) and after fermentation. Values with different lower-case letters are significantly different among all samples (*p* < 0.05).

**Figure 4:**
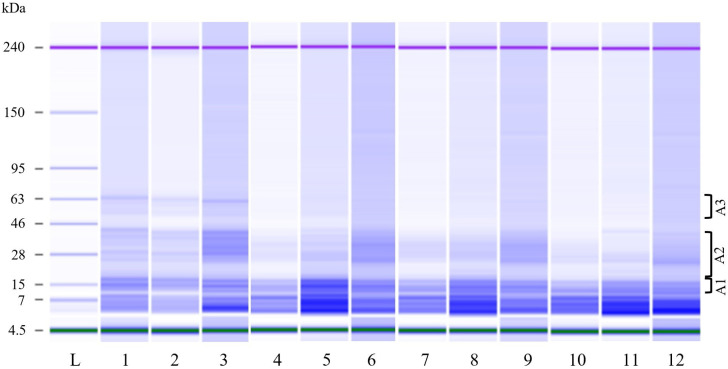
Protein profile of total proteins extracted from sourdough formulations before (T0) (CTR_T0) and after (T14) fermentation (C43-11, C43-2M and ITM21B). Lane L, sizing ladder; CTRs (T0): O (1), F (2), S (3); O_C43-11 (4), F_C43-11 (5) and S_C43-11 (6); O_C43-2M (7), F_C43-2M (8) and S_C43-2M (9); O_ITM21B (10), F_ITM21B (11) and S_ITM21B (12); brackets indicate molecular weight areas as reported in paragraph 2.7.2.

**Figure 5:**
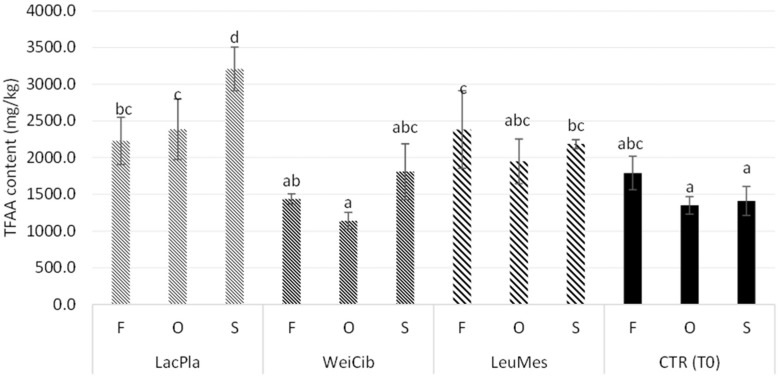
Total free amino acids content in sourdough formulations, before (CTR_T0) and after fermentation. Values with different lower-case letters are significantly different among all samples (*p* < 0.05).

Slight differences were observed in the content and profile of total proteins (Figure 3 and Figure 4). The total protein content mainly decreased in the presence of the *L. plantarum* strain in S_LacPla, although without a significant difference. The results were consistent with the protein profile observed after fermentation (Figure 4). In fact, the HMw proteins were mainly transformed into smaller proteins (Figure 4) and amino acids (Figure 5). 

The electrophoretic patterns of total proteins extracted from sourdoughs based on each okra flour at the beginning (CTRs) and after 14 h of fermentation (T14) are reported in Figure 4. At the beginning of fermentation, the banding patterns of the three okra flours were similar and characterized by about 11 protein bands in the range of 14–64 kDa distributed in Mw A1 (43.9 ± 11.8%), Mw A2 (46.4 ±14.9%) and Mw A3 (9.8 ± 3.1%); major peaks/bands (43.9% of the total) were displayed at 14.8 ± 0.2 and 17 ± 0.2 kDa and at 32.9 kDa only in S_CTR_T0 (17.9% of the total). After 14 h of fermentation, modified protein patterns were observed in all sourdough samples, showing about eight protein peaks/bands in the 14–41 kDa range and the complete disappearance of high molecular weight proteins in Mw A3; the main peaks, accounting for 48.7% of the total, were still present at 14.2 ± 0.1 and 16.5 ± 0.1 kDa. Although the protein patterns of sourdoughs fermented by strains C43-11 and C43-2M were similar, a different increase in the percentage of peaks/bands in Mw areas was observed depending on the strain and flour type: in F_WeiCib and F_LeuMes sourdoughs, a 43.6% and a 27.1% increase in peaks/bands in Mw A1 (14–30 kDa) was observed, whereas in O_WeiCib and O_LeuMes sourdoughs, a 24.7% and 33.5% increase in peaks/bands in Mw A2 (21–42 kDa), respectively, was detected; in sourdoughs based on S flour, slight increases were observed in both areas and with both strains. When O flour was fermented with the *L. plantarum* ITM21B strain, a higher proteolysis occurred than in the corresponding CTR, as six protein peaks/bands were distributed in the 14–36 kDa range and a 24.9% increase in percentage of peaks/bands in Mw A2 was observed; in F_LacPla and S_LacPla, eight protein peaks/bands ranging from 14 to 41 kDa were visualized with an overall 25% increase in the percentage of peaks/bands in Mw A1.

Samples fermented by the *L. plantarum* ITM21B strain, mainly sourdoughs containing O and S flours, showed the most significant increase in TFAA content when compared to CTR samples (Figure 5). In fact, fermentation induced the increase in TFAA mainly in S_LacPla, O_LacPla, F_LeuMes and S_LeuMes. These compounds are fundamental for the nutritional value of the product and for its flavor [57].

Considering the small amount of okra flour used to prepare sourdough, the changes observed in the metabolite composition after fermentation suggested the potential impact that this vegetable could exert on the quality of the food product. Data from the fermentation test highlighted that organic acids, which play an important role in dough acidification and in the subsequent activation of cereal enzymes and antimicrobial properties, were produced differently in the sourdough formulations. In particular, lactic and propionic acid production was mainly registered in sourdough started with *L. plantarum* with all okra flours, whereas acetic acid was only produced by *Leuc. mesenteroides* and *W. cibaria* in all sourdough formulations. EPS, metabolites that could have a role in the technological properties of the final product, were mainly produced after fermentation in O_LeuMes sourdough, while the l-glutamate, a taste-active compound that can be used in the substitution of salt, was produced in almost all sourdough samples. The total protein content indicated the occurrence of proteolysis during fermentation, mainly related to the conversion of HMw proteins into smaller ones and into amino acids. Although no significant decrease in total protein content was observed after fermentation, the electrophoretic pattern highlighted a modification of the protein profile with the production of small proteins mainly in *L. plantarum* sourdough. Finally, in the case of TFAA, associated to the flavor characteristics of bread, a higher production after fermentation was observed in the O_LacPla, S_LacPla and S_WeiCib sourdough samples. To detect any effect of okra flour addition, the nature of the starter and dehydration process on the quality parameters monitored in the current study, Principal Component Analysis was performed.

### 3.2. Principal Component Analysis 

**Figure 6:**
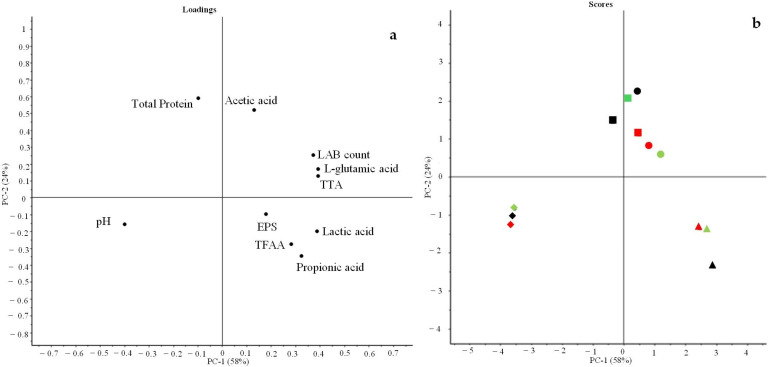
Principal component analysis (PCA) of all data registered on sourdough. (**a**) Loading plot indicating the correlation between variables (pH; TTA; LAB count; EPS; l-glutamic acid; organic acids—lactic; acetic; propionic; total proteins; TFAA content). (**b**) Score plot indicating sample distribution basing on the inoculated strains (triangle LacPla; circle LeuMes; square WeiCib; diamond CTRs) used to ferment F (red symbol), O (green symbol) and S (black symbol) okra flours.

To better discriminate the sourdough samples, PCA was performed on all data (pH, TTA, EPS, organic acids, total protein content, TFAA, l-glutamic acid) (Figure 6). Figure 6a (loading plot) and the correlation analysis (Table 4) clearly show the negative correlation between the content of total protein and that of small molecules related to the secondary metabolism, such as TFAA. Conversely, the higher levels of TTA were positively correlated with the higher protein content and then to the absence of proteolysis. A positive correlation was observed between lactic acid and all parameters except for total protein content and pH, indicating the key role of this compound in sourdough metabolism. As shown in the score plot (Figure 6b), all control samples (before fermentation) were located in the lower left part of the graph, corresponding to the higher pH values as initial conditions. Fermented okra flours were discriminated mainly on the basis of the starter strain. Sourdoughs fermented by *W. cibaria* C43-11 and *Leuc. mesenteroides* C43-2M were more similar in composition with only minor differences between samples. The F_WeiCib, F_LeuMes and O_LeuMes sourdoughs were grouped in the right part of the graph because they were characterized by higher LAB counts, l-glutamic acid contents and TTA values. On the other hand, the acetic acid content mainly distinguished S_LeuMes and O_WeiCib from the other samples. In the lower right part of the graph, the sourdoughs inoculated with *L. plantarum* ITM21B strain were located and distinguished by the lower protein content, higher TFAA, EPS, and propionic and lactic acids. 

Among the sourdough formulations, since only small differences were observed between the samples and the oven-drying technology is easily applicable in the food sector, the sourdough based on the starter *Leuc. mesenteroides* C43-2M and O flour was selected for the baking test, to further investigate the potential of this formulation in the production of bakery products. 

### 3.3. Bread Properties 

**Figure 7:**
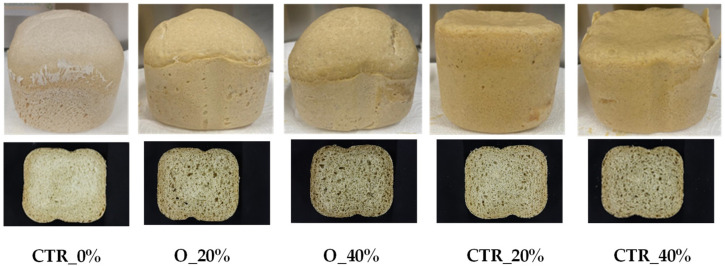
Bread samples prepared using fermented or unfermented oven-dried okra flour (O). CTR_0%, standard recipe; bread O_20%, bread O_40% (*w/w* flour weight), both containing O_LeuMes corresponding to 0.6% and 1.21% (*w/w*) of O flour replacement; breads CTR_20% and CTR_40% (containing not fermented O flour at 0.6% and 1.21% *w/w*, respectively).

The fermented sourdough prepared with *Leuc. mesenteroides* C43-2M, the O flour and the unfermented O flour were used to prepare bread prototypes. The general appearance of the bread samples is shown in Figure 7. The images show the suitability of sourdough as an ingredient for yeast-leavened bread making. Table 5 reports the specific volume of the bread loaf and the moisture content, a_w_, color and firmness of the crumb. The inclusion of O_LeuMes sourdough in the bread formulation did not significantly modify the moisture content of the crumb, with only minor changes in a_w_. The latter was probably due to differences in batch preparation rather than a specific effect of the presence of okra flour. As expected, despite the small amount of okra flour used in the formulation (0.6% and 1.21% O flour replacement), its presence led to significant color changes in the crumb, with a decrease in the whiteness/lightness L* value and an increase in redness a* values, as also shown by the ΔE values. This darkening effect was independent from the occurrence of fermentation and was attributed to the brown color of the O flour. Previous studies have reported the strong impact of oven-dried okra flour on bread color [5,18,20,60].

It is noteworthy that the presence of okra induced a reduction in specific volume with a concomitant increase in firmness. This was particularly evident in the bread sample containing unfermented okra flour at the higher content (1.21%). This behaviour can be attributed to the presence of okra components with high water binding capacity (e.g., pectin and other polysaccharides). These components may compete with other ingredients for water, reducing the availability of water for gluten–starch network formation and thus reducing dough elasticity, gas entrapment and leavening capacity. This is known from the literature for other plant fibers [21,62,63,64,65,66]. Our data on volume and firmness are consistent with the literature. Alamri [64] tested the effect of freeze-dried okra extract incorporated into bread at 4, 7, 10 and 13%: firmness increased and color became darker as a function of okra flour replacement. Recently, Bai-Ngew et al. [20] investigated the effect of two okra powders obtained by microwave vacuum or a hot air-drying process, incorporated in bread formulation at 1, 3 and 5%. The results indicated that the specific volume of the bread was inversely correlated with the addition of okra. The exception was the 1% replacement, which resulted in an improvement in specific volume, the bread’s hardness and bioactive compound content. 

As a naturally gluten-free (GF) material, okra flour can also be a valuable ingredient in GF bread. Interesting results were obtained by Tufaro et al. [18] who produced oven-dried okra powder (whole and fine) and incorporated it into the GF bread recipe at 1.5% of dough weight in combination with hydrocolloids. The authors observed that okra powders mainly affected the amount of water needed to achieve the desired dough consistency and, to a lesser extent, the color of the final product, while the technological traits were improved by the type of hydrocolloid used (hydroxypropyl methylcellulose rather than *Psyllium* fiber). 

**Figure 8:**
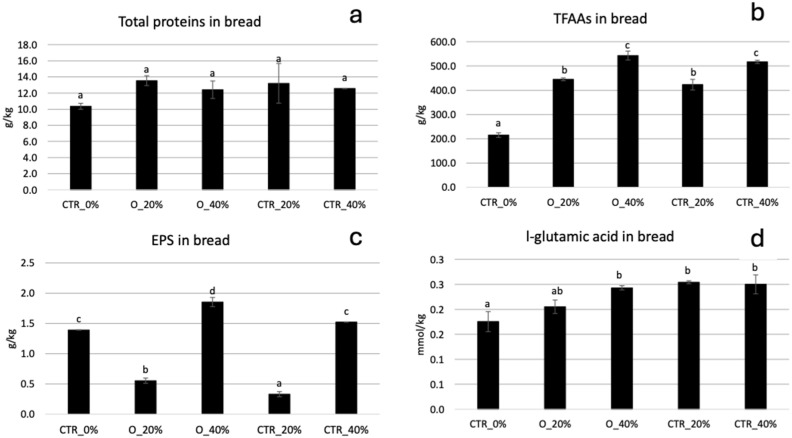
Total proteins (**a**), TFAAs (**b**), EPS (**c**) and l-glutamic acid (**d**) content in bread samples prepared using unfermented (CTR_20% and CTR_40%) O flour or O_LeuMes sourdough (O_20% and O_40%). CTR_0% is the standard bread without okra flour. Values with different lower-case letters are significantly different among all samples (*p* < 0.05).

**Figure 9:**
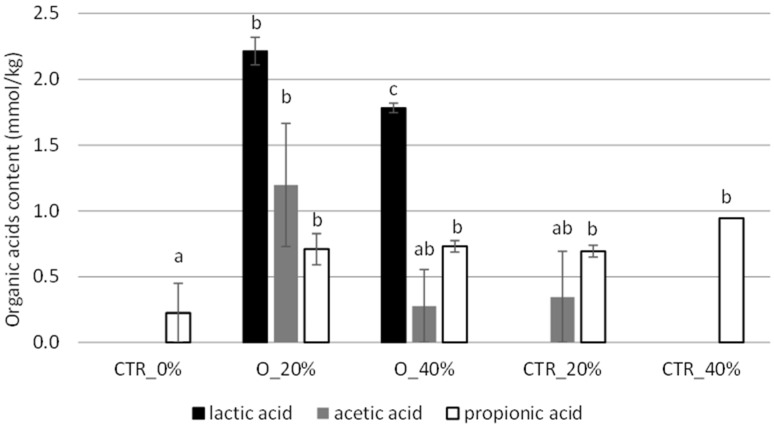
Organic acids (lactic, acid and propionic) content in bread samples prepared using unfermented (CTR_20% and CTR_40%) or O_LeuMes sourdough (O_20% and O_40%). CTR_0% is the standard bread without okra flour. Values of each acid with different lower-case letters are significantly different among all samples (*p* < 0.05).

In our study, the partial replacement of wheat flour with unfermented O flour at 0.6% and 1.21% had some negative effects on bread quality as compared to the CTR_0% not containing okra flour, while the fermentation process improved the specific volume and reduced the firmness of bread containing the higher amount of sourdough (40% corresponding to the 1.21% flour replacement) with respect to the CTR bread containing unfermented O flour at the same level (Table 5). Actually, even if these are preliminary results and a better understanding of the effects of okra flour type and percentage replacement on bread characteristics there is needed, it seems that fermentation partially reduced these detrimental effects, especially considering the O_40% bread This effect could be due to the microbial activity leading to acidification of the dough and hydrolysis of okra components as well as to the slightly higher content of EPS (Figure 8 and Figure 9). 

As reported by Nawab et al. [67], a polysaccharide such as dextran, which has a high molecular weight and linear chain structure binds moisture, then acting as a plasticizer that can limit starch swelling and gelatinization, thereby delaying the staling process and prolonging shelf life. Dextran, a homopolysaccharide belonging to the exopolysaccharide compounds produced by LAB, is frequently recovered in sourdough and is characterized by several technological and health properties [29,68,69]. In particular, it can act as a hydrocolloid in bread formulation, contributing to the improvement in the product’s texture and sensory qualities, as well as meeting consumer demands for clean-label and healthy foods [70]. Dough quality is highly dependent on gluten solubility, which increases in an acidic environment. The presence of polysaccharides with a high water holding capacity leads to increased formation of β-structures, aggregation and abnormal folding of gluten proteins [71]. Bi et al. [59] demonstrated the influence of the acidic environment on the interaction between HMw dextran and gluten structure as the depolymerization of gluten aggregates caused by HMw dextran was weakened under acidic conditions (pH = 3.6), while it was promoted in a weakly acidic environment (pH = 4.4–4.6). These results confirm the positive role that polysaccharides can play in combination with a controlled LAB fermentation.

The EPS content in bread was in agreement with other published data [72]. In our study, the main aim of quantifying EPS was to compare breads containing sourdough with those containing unfermented okra flour. As shown in Figure 8, significant differences in the content of EPS were registered among the samples, with the highest value observed in bread containing the fermented O_LeuMes sourdough at 40%. The microbial contribution to the polysaccharidic fraction in bread can be hypothesized since the amount of these compounds in both sourdough-containing breads (O_20% and O_40%) was higher than controls containing unfermented okra flour (CTR_20% and CTR_40%). The higher amount of polysaccharides in CTR_40% than CTR_20% could be due to the higher concentration of okra flour. The differences between control samples could be related to the presence of starch, mainly in CTR_0%, which led to an increase in the carbohydrate content as quantified by the phenol-sulfuric acid method. 

The content of l-glutamic acid was significantly higher in samples containing sourdough and unfermented O flour, suggesting the contribution of okra flour to the content of this amino acid. Actually, glutamic acid is one of the most abundant amino acids in okra pods and particularly in seeds [73]. The protein content did not vary between samples since okra pods are not particularly rich in these compounds while containing many amino acids [3]. In fact, the amount of TFAAs was significantly higher in sourdough-based bread samples. Among the organic acids, a higher level was observed in the samples produced with O flour compared to the standard bread not containing okra flour (CTR_0%) (Figure 9). In particular, the lactic acid level was significantly higher in bread produced with the sourdough, while the other acids were found to be at very low levels in all samples.

In order to better understand the correlations between technological and analytical measures registered on bread, Pearson correlation coefficients were calculated. The results are presented in Table 6 and clearly show the significant correlation between total protein content, TFAA, pH, TTA and almost all the technological parameters. All analytical parameters, except for pH, were negatively correlated with specific volume, a_w_ and lightness L*, and positively correlated with firmness, moisture, redness a* and yellowness b*, suggesting the influence of the microbial metabolism characterizing sourdough on bread quality attributes. In detail, specific volume was positively correlated with pH and negatively correlated with all the other parameters. Actually, the acidification of bread (low pH and high TTA) can have a negative effect on the specific volume and firmness of bread due to the breakdown of the gluten protein [16]. 

## 4. Conclusions

The data obtained in the current study suggest that the valorization of okra pod flours by fermentation in the sourdough process represents a biotechnological strategy that can ameliorate the technological disadvantages caused by the use of unprocessed okra flour in bread making, paving the way for the production of innovative fermented food ingredients based on this precious vegetable. The fermentation process allowed the content of exopolysaccharides (EPS), total free amino acids (TFAA), l-glutamic acid and organic acids to be increased in the sourdough and the incorporation of okra flour into the bread-making process to be simplified. Bread containing the *Leuconostoc mesenteroides* wheat-based sourdough with 9% oven-dried okra flour added at 40% *w/w* in its formulation, corresponding to a replacement level of okra flour of 1.21%, was improved in nutritional and technological properties, mainly for the content of TFAA, EPS and l-glutamic acid, and showed an improved specific volume and firmness with respect to the unfermented oven-dried okra flour bread. 

## Figures and Tables

**Table 1:** Chemical composition of produced okra flours.

	F	O	S
Moisture (g/100 g)	0.22 ± 0.02	9.85 ± 0.1	0.00 ± 0.0
Protein content (g/100 g db ^#^)	18.01 ± 0.25	19.04 ± 0.18	18.39 ± 0.15
Total fats (g/100 g db)	18.26 ± 0.36	2.40 ± 0.276	1.18± 0.08
Total carbohydrates (g/100 g db)	26.71 ± 0.26	35.27 ± 0.37	24.88 ± 3.34

^#^ db, dry basis. Flour codes are reported in Section 2.1. Data are presented as mean ± standard deviation.

**Table 2:** Formulations of bread containing O_LeuMes sourdough at two different percentages in comparison to controls.

	Bread Variant *				
	CTR_0%	O_20%	O_40%	CTR_20%	CTR_40%
Ingredients	Weight (g)				
Wheat flour	350	326.67	303.33	347.88	345.76
Salt	6.3	6.3	6.3	6.3	6.3
Olive oil	10.5	10.5	10.5	10.5	10.5
Dry yeast	5.25	5.25	5.25	5.25	5.25
Tap water	210	163.33	116.67	210	210
O_LeuMes	-	70	140	-	-
O flour	-	-	-	2.12	4.24

* O_20%, bread containing O_LeuMes sourdough at 20% sourdough *w/w* flour; O_40%, bread containing O_LeuMes sourdough at 40% sourdough *w/w* flour; CTR_0%, not containing O flour; CTR_20%, bread containing unfermented O flour (corresponding to okra flour content in O_20%); CTR_40%, bread containing unfermented O flour (corresponding to okra flour content in O_40%).

**Table 3:** LAB load, pH and TTA values before (CTR) and after fermentation in the sourdough formulations based on O, F and S flours.

Okra Flour Type	Sourdough	LAB (log CFU/g) ± SE	pH ± SE	TTA (mL NaOH 0.1 N) ± SE
Oven-dried	O_CTR_T0	5.16 ± 0.26 b	5.92 ± 0.02 a	1.00 ± 0.10 a
	O_WeiCib	9.33 ± 0.08 cd	4.03 ± 0.02 c	4.75 ± 0.25 bc
	O_LeuMes	9.43 ± 0.16 cd	3.96 ± 0.02 c	4.45 ± 0.55 b
	O_LacPla	9.26 ± 0.23 cd	3.57 ± 0.01 d	6.50 ± 0.50 d
Freeze-dried	F_CTR_T0	3.15 ± 0.15 a	6.00 ± 0.02 a	0.65 ± 0.15 a
	F_WeiCib	9.45 ± 0.26 cd	4.02 ± 0.01 c	4.65 ± 0.35 bc
	F_LeuMes	9.57 ± 0.33 cd	4.00 ± 0.07 c	4.50 ± 0.50 b
	F_LacPla	9.74 ± 0.03 d	3.70 ± 0.12 d	6.00 ± 1.00 cd
Sun-dried	S_CTR_T0	3.15 ± 0.15 a	5.62 ± 0.04 b	0.95 ± 0.15 a
	S_WeiCib	9.17 ± 0.13 c	3.95 ± 0.09 c	3.45 ± 0.55 b
	S_LeuMes	9.16 ± 0.09 c	3.98 ± 0.06 c	4.75 ± 0.25 bc
	S_LacPla	9.26 ± 0.06 cd	3.55 ± 0.00 d	4.40 ± 0.10 b

Within each column, values (mean ± standard error of the mean) with different lower-case letters are significantly different (*p* < 0.05).

**Table 4:** Pearson correlation coefficients among analytical data (EPS, organic acid content, l-glutamic acid, total proteins, TFAA, pH and TTA) registered on sourdough samples.

	pH	TTA	LAB Count	EPS	Lactic Acid	Acetic Acid	Propionic Acid	L-Glutamic Acid	Total Proteins	TFAA
pH	-	*−0.94*	*−0.95*	−0.26	*−0.76*	*−0.44*	−0.52	*−0.70*	0.00	*−0.49*
TTA	*−0.94*	-	*0.91*	0.17	*0.75*	*0.33*	0.57	*0.72*	0.04	*0.40*
LAB count	*−0.95*	*0.91*	-	0.17	*0.65*	*0.51*	0.44	*0.70*	0.09	*0.37*
EPS	−0.26	0.17	0.17	-	*0.29*	0.15	*0.33*	0.07	−0.16	*0.32*
lactic acid	*−0.76*	*0.75*	*0.65*	*0.29*	-	0.11	*0.76*	*0.32*	*−0.41*	*0.68*
acetic acid	*−0.44*	*0.33*	*0.51*	0.15	0.11	-	−0.01	*0.30*	0.17	0.16
propionic acid	*−0.52*	*0.57*	*0.44*	*0.33*	*0.76*	−0.01	-	0.19	*−0.40*	*0.58*
l-glutamic acid	*−0.70*	*0.72*	*0.70*	0.07	*0.32*	*0.30*	0.19	-	0.22	0.11
total proteins	0.00	0.04	0.09	−0.16	*−0.41*	0.17	*−0.40*	0.22	-	*−0.37*
TFAA	*−0.49*	*0.40*	*0.37*	*0.32*	*0.68*	0.16	*0.58*	0.11	*−0.37*	-

Values in italic character indicate significant correlation (*p* < 0.05).

**Table 5:** Moisture, a_w_, color and firmness of crumb and loaf specific volume of bread samples prepared using unfermented O flour (CTR_20% and CTR_40%) or O_LeuMes sourdough (O_20% and O_40%). CTR_0% is the standard bread without okra flour. Values with different lower-case letters are significantly different among all samples (*p* < 0.05).

Bread Samples	Specific Volume (cm^3^/g)	Moisture	a_w_	L*	a*	b*	ΔE	Firmness (N)
CTR_0%	4.08 ± 0.81 a	66.87 ± 0.90 a	0.64 ± 0.01 a	71.48 ± 3.16 a	−1.44 ± 0.15 d	15.21 ± 0.81 a		1.13 ± 0.81 c
O_20%	2.30 ± 0.81 b	68.86 ± 0.71 a	0.63 ± 0.01 ab	62.29 ± 2.71 b	−1.18 ± 0.20 c	15.48 ± 0.99 a	9.92	1.87 ± 0.07 b
O_40%	2.44 ± 0.81 b	65.87 ± 1.46 a	0.61 ± 0.02 bca	57.65 ± 2.17 c	−0.69 ± 0.13 a	15.23 ± 0.92 a	13.85	1.75 ± 0.03 b
CTR_20%	2.38 ± 0.81 b	65.66 ± 1.13a	0.60 ± 0.01 bc	62.58 ± 2.52 b	−1.00 ± 0.11 bc	15.73 ± 0.74 a	8.92	1.98 ± 0.14 b
CTR_40%	1.75 ± 0.81 c	68.74 ± 1.78 a	0.58 ± 0.02 c	60.77 ± 2.13 bc	−0.85 ± 0.11 ab	16.10 ± 0.54 a	10.76	3.01 ± 0.11 a

**Table 6:** Pearson correlation coefficients between technological (color, moisture, a_w_, firmness of crumb and loaf specific volume) and analytical (EPS, organic acids content, l-glutamic acid, total proteins, TFAA, pH and TTA) data relevant to bread.

		Analytical Data								
		EPS	Lactic Acid	Acetic Acid	Propionic Acid	L-Glutamic Acid	Total Proteins	TFAA	pH	TTA
Technological data	L*	−0.07	−0.18	−0.11	*−0.93*	*−0.79*	*−0.58*	*−0.88*	*0.60*	*−0.30*
	a*	*0.34*	*0.28*	0.05	*0.87*	*0.64*	*0.43*	*0.87*	*−0.50*	*0.41*
	b*	0.09	*0.42*	0.18	0.09	0.14	*0.30*	*0.28*	*−0.44*	*0.50*
	moisture	0.07	−0.06	*−0.27*	−0.09	*0.40*	0.00	0.13	*−0.54*	0.17
	a_w_	*−0.47*	*−0.74*	−0.24	*−0.54*	*−0.36*	*−0.37*	*−0.79*	*0.71*	*−0.89*
	firmness	*0.33*	*0.65*	0.19	*0.48*	*0.54*	*0.41*	*0.79*	*−0.90*	*0.85*
	specific volume	−0.01	*−0.52*	*−0.30*	*−0.80*	*−0.79*	*−0.72*	*−0.94*	*0.95*	*−0.66*

Values in italic character indicate significant correlation (*p* < 0.05).

## Data Availability

The original contributions presented in the study are included in the article, further inquiries can be directed to the corresponding author.

## Abbreviations

**S.No**	**Notation**	**Description**
1	O	oven-dried okra flour
2	F	freeze-dried okra flour
3	S	sun-dried okra flour
4	DY	dough yield
5	CTR	control
6	O_CTR_T0	not inoculated mixtures, containing O flour, before fermentation
7	O_WeiCib	sourdough inoculated with *W. cibaria* C43-11 strain, containing O flour, after 14 h of fermentation
8	O_LeuMes	sourdough inoculated with *Leuc. mesenteroides* C43-2M strain, containing O flour, after 14 h of fermentation
9	O_LacPla	sourdough inoculated with *L. plantarum* ITM21B strain, containing O flour, after 14 h of fermentation
10	F_CTR_T0	not inoculated mixtures, containing F flour, before fermentation
11	F_LeuMes	sourdough inoculated with *W. cibaria* C43-11 strain, containing F flour, after 14 h of fermentation
12	F_LeuMes	sourdough inoculated with *Leuc. mesenteroides* C43-2M strain, containing F flour, after 14 h of fermentation
13	F_LacPla	sourdough inoculated with *L. plantarum* ITM21B strain, containing F flour, after 14 h of fermentation
14	S_CTR_T0	not inoculated mixtures, containing S flour, before fermentation
15	S_WeiCib	sourdough inoculated with *W. cibaria* C43-11 strain, containing S flour, after 14 h of fermentation
16	S_LeuMes	sourdough inoculated with *Leuc. mesenteroides* C43-2M strain, containing S flour, after 14 h of fermentation
17	S_LacPla	sourdough inoculated with *L. plantarum* ITM21B strain, containing S flour, after 14 h of fermentation
18	Mw	molecular weight
19	HMw	high molecular weight
20	GF	gluten-free
21	CTR_0%	bread not containing okra flour, produced with standard recipe
22	O_20%	bread containing 20% of O_LeuMes (*w/w* flour weight), corresponding to 0.6% (*w/w*) of O flour replacement
23	O_40%	bread containing 40% of O_LeuMes (*w/w* flour weight), corresponding to 1.21% (*w/w*) of O flour replacement
24	CTR_20%	bread containing not fermented O flour at 0.6% *w/w*
25	CTR_40%	bread containing not fermented O flour at 1.21% *w/w*

## References

[1] Islam M.T. (2019). Phytochemical information and pharmacological activities of Okra (*Abelmoschus esculentus*): A literature-based review. Phytother. Res..

[2] Wu D.T., Nie X.R., Shen D.D., Li H.Y., Zhao L., Zhang Q., Lin D.R., Qin W. (2020). Phenolic compounds, antioxidant activities, and inhibitory effects on digestive enzymes of different cultivars of okra (*Abelmoschus esculentus*). Molecules.

[3] Romdhane M.H., Chahdoura H., Barros L., Dias M.I., Corrêa R.C.G., Morales P., Ciudad-Mulero M., Flamini G., Majdoub H., Ferreira I.C. (2020). Chemical composition, nutritional value, and biological evaluation of Tunisian okra pods (*Abelmoschus esculentus* L. Moench). Molecules.

[4] Adelakun O.E., Oyelade O.J., Preedy V.R., Watson R.R., Patel V.B. (2011). Potential use of okra seed (*Abelmoschus esculentus* Moench) flour for food fortification and effects of processing. Flour and Breads and Their Fortification in Health and Disease Prevention.

[5] Machine A.P., Massingue A.A., Asaam S. (2020). Physicochemical and sensory properties of bread with sweet potato flour (*Ipomea batatas* L.) as partial replacer of wheat flour supplemented with okra hydrocolloids. Afr. J. Food Sci..

[6] Gemede H.F., Ratta N., Haki G.D., Woldegiorgis A.Z., Beyene F. (2015). Nutritional quality and health benefits of okra (*Abelmoschus esculentus*): A review. Int. J. Nutr. Food Sci..

[7] Nie X.R., Fu Y., Wu D.T., Huang T.T., Jiang Q., Zhao L., Zhang Q., Lin D.R., Chen H., Qin W. (2020). Ultrasonic-assisted extraction, structural characterization, chain conformation, and biological activities of a pectic-polysaccharide from okra (*Abelmoschus esculentus*). Molecules.

[8] Nie X.R., Li H.Y., Du G., Lin S., Hu R., Li H.Y., Zhao L., Zhang Q., Chen H., Wu D.T. (2019). Structural characteristics, rheological properties, and biological activities of polysaccharides from different cultivars of okra (*Abelmoschus esculentus*) collected in China. Int. J. Biol. Macromol..

[9] Maalej H., Maalej A., Bayach A., Zykwinska A., Colliec-Jouault S., Sinquin C., Marchand L., Ktari N., Bardaa S., Salah R.B. (2023). A novel pectic polysaccharide-based hydrogel derived from okra (*Abelmoschus esculentus* L. Moench) for chronic diabetic wound healing. Eur. Polym. J..

[10] Liu H., Gong F., Wei F., Wu H. (2018). Artificial simulation of salivary and gastrointestinal digestion, and fermentation by human fecal microbiota, of polysaccharides from *Dendrobium aphyllum*. RSC Adv..

[11] Xia F., Zhong Y., Li M., Chang Q., Liao Y., Liu X., Pan R. (2015). Antioxidant and anti-fatigue constituents of okra. Nutrients.

[12] Yuan Q., Lin S., Fu Y., Nie X.R., Liu W., Su Y., Han Q.H., Zhao L., Zhang Q., Lin D.R. (2019). Effects of extraction methods on the physicochemical characteristics and biological activities of polysaccharides from okra (*Abelmoschus esculentus*). Int. J. Biol. Macromol..

[13] Wu D.T., Fu Y., Guo H., Yuan Q., Nie X.R., Wang S.P., Gan R.Y. (2021). In vitro simulated digestion and fecal fermentation of polysaccharides from loquat leaves: Dynamic changes in physicochemical properties and impacts on human gut microbiota. Int. J. Biol. Macromol..

[14] Sengkhamparn N., Bakx E.J., Verhoef R., Schols H.A., Sajjaanantakul T., Voragen A.G. (2009). Okra pectin contains an unusual substitution of its rhamnosyl residues with acetyl and alpha-linked galactosyl groups. Carbohydr. Res..

[15] Manzocco L., Basso F., Nicoli M.C. (2023). Effect of hyperbaric storage at room temperature on the activity of polyphenoloxidase in model systems and fresh apple juice. Food Bioprocess Technol..

[16] Islam M.A., Islam S. (2024). Sourdough bread quality: Facts and Factors. Foods.

[17] Padayachee A., Netzel G., Netzel M., Day L., Zabaras D., Mikkelsen D., Gidley M.J. (2012). Binding of polyphenols to plant cell wall analogues–Part 2: Phenolic acids. Food Chem..

[18] Tufaro D., Bassoli A., Cappa C. (2022). Okra (*Abelmoschus esculentus*) powder production and application in gluten-free bread: Effect of particle size. Food Bioprocess Technol..

[19] Machine A.P., Massingue A.A., Balane S.G., Armando E.J., Son L. (2024). Enhancing bread quality: Investigating the physicochemical and sensory properties of bread with sweet potato flour and okra hydrocolloids as substitutes for wheat flour. Current Perspectives in Agriculture and Food Science.

[20] Bai-Ngew S., Phimolsiripol Y., Walter P. (2024). Characterization of hot air drying and microwave vacuum drying of okra powder and application in bread. Food Appl. Biosci. J..

[21] Xu K., Guo M., Roman L., Pico J., Martinez M.M. (2020). Okra seed and seedless pod: Comparative study of their phenolics and carbohydrate fractions and their impact on bread-making. Food Chem..

[22] Ou J., Wang M., Zheng J., Ou S. (2019). Positive and negative effects of polyphenol incorporation in baked foods. Food Chem..

[23] Li M., Koecher K., Hansen L., Ferruzzi M.G. (2017). Phenolics from whole grain oat products as modifiers of starch digestion and intestinal glucose transport. J. Agric. Food Chem..

[24] Pico J., Martinez M.M. (2019). Unraveling the inhibition of intestinal glucose transport by dietary phenolics: A review. Curr. Pharm. Des..

[25] Pico J., Corbin S., Ferruzzi M.G., Martinez M.M. (2019). Banana flour phenolics inhibit trans-epithelial glucose transport from wheat cakes in a coupled in vitro digestion/Caco-2 cell intestinal model. Food Funct..

[26] Alamri M.S. (2014). Okra-gum fortified bread: Formulation and quality. J. Food Sci. Technol..

[27] Buczkowski B.K. (2013). Sourdough Bread Enriched with Soluble Fibres: Development, Characterisation and Nutritional Aspects of a Functional Food Product. Doctoral Dissertation.

[28] De Vuyst L., Van Kerrebroeck S., Harth H., Huys G., Daniel H.-M., Weckx S. (2014). Microbial ecology of sourdough fermentations: Diverse or uniform?. Food Microbiol..

[29] Arendt E.K., Ryan L.A., Dal Bello F. (2007). Impact of sourdough on the texture of bread. Food Microbiol..

[30] Ripari V., Bai Y., Gänzle M.G. (2019). Metabolism of phenolic acids in whole wheat and rye malt sourdoughs. Food Microbiol..

[31] Gabriele M., Sparvoli F., Bollini R., Lubrano V., Longo V., Pucci L. (2019). The impact of sourdough fermentation on non-nutritive compounds and antioxidant activities of flours from different *Phaseolus vulgaris* L. genotypes. J. Food Sci..

[32] Altunkaya A., Hedegaard R.V., Brimer L., Gökmen V., Skibsted L.H. (2013). Antioxidant capacity versus chemical safety of wheat bread enriched with pomegranate peel powder. Food Funct..

[33] Gänzle M.G. (2009). From gene to function: Metabolic traits of starter cultures for improved quality of cereal foods. Int. J. Food Microbiol..

[34] Korakli M., Rossmann A., Gänzle M.G., Vogel R.F. (2001). Sucrose metabolism and exopolysaccharide production in wheat and rye sourdoughs by *Lactobacillus sanfranciscensis*. J. Agr. Food Chem..

[35] Adelakun O.E., Olanipekun B.F., Akingbaso O., Adhikar B.M. (2017). Effect of fermentation and variety on quality attributes of okra seed (*Abelmoschus esculentus* (L.) Moench) flour. Donn. J. Food Sci. Tech..

[36] Adetuyi F.O., Ibrahim T.A. (2014). Effect of fermentation time on the phenolic, flavonoid and vitamin C contents and antioxidant activities of okra (*Abelmoschus esculentus*) seeds. Niger. Food J..

[37] Wang X., Hu K., Chen Y., Lai J., Zhang M., Li J., Li Q., Zhao N., Liu S. (2024). Effect of *Lactiplantibacillus plantarum* fermentation on the physicochemical, antioxidant activity and immunomodulatory ability of polysaccharides from Lvjian okra. Int. J. Biol. Macromol..

[38] Corsetti A., Lavermicocca P., Morea M., Baruzzi F., Tosti N., Gobbetti M. (2001). Phenotypic and molecular identification and clustering of lactic acid bacteria and yeasts from wheat (species *Triticum durum* and *Triticum aestivum*) sourdoughs of southern Italy. Int. J. Food Microbiol..

[39] Di Biase M., Bavaro A.R., Lonigro S.L., Pontonio E., Conte A., Padalino L., Minisci A., Lavermicocca P., Valerio F. (2019). *Lactobacillus plantarum* ITM21B fermentation product and chickpea flour enhance the nutritional profile of salt reduced bakery products. Int. J. Food Sci. Nutr..

[40] Valerio F., Conte A., Di Biase M., Lattanzio V.M., Lonigro S.L., Padalino L., Pontonio E., Lavermicocca P. (2017). Formulation of yeast-leavened bread with reduced salt content by using a *Lactobacillus plantarum* fermentation product. Food Chem..

[41] De Bellis P., Ferrara M., Bavaro A.R., Linsalata V., Di Biase M., Musio B., Gallo V., Valerio F. (2022). Characterization of dextran produced by the food-related strain *Weissella cibaria* C43-11 and of the relevant dextransucrase gene. Foods.

[42] Valerio F., Favilla M., De Bellis P., Sisto A., de Candia S., Lavermicocca P. (2009). Antifungal activity of strains of lactic acid bacteria isolated from a semolina ecosystem against *Penicillium roqueforti*, *Aspergillus niger* and *Endomyces fibuliger* contaminating bakery products. Syst. Appl. Microbiol..

[43] Bavaro A.R., Di Biase M., Conte A., Lonigro S.L., Caputo L., Cedola A., Del Nobile A., Logrieco A.F., Lavermicocca P., Valerio F. (2021). *Weissella cibaria* short-fermented liquid sourdoughs based on quinoa or amaranth flours as fat replacer in focaccia bread formulation. Int. J. Food Sci. Technol..

[44] Valerio F., Bavaro A.R., Di Biase M., Lonigro S.L., Logrieco A.F., Lavermicocca P. (2020). Effect of amaranth and quinoa flours on exopolysaccharide production and protein profile of liquid sourdough fermented by *Weissella cibaria* and *Lactobacillus plantarum*. Front. Microbiol..

[45] Koutsoumanis K., Allende A., Álvarez-Ordóñez A., Bolton D., Bover-Cid S., Chemaly M., De Cesare A., Hilbert F., Lindqvist R., EFSA Panel on Biological Hazards (BIOHAZ) (2023). Scientific Opinion on the update of the list of qualified presumption of safety (QPS) recommended microorganisms intentionally added to food or feed as notified to EFSA. EFSA J..

[46] De Bellis P., Valerio F., Sisto A., Lonigro S.L., Lavermicocca P. (2010). Probiotic table olives: Microbial populations adhering on olive surface in fermentation sets inoculated with the probiotic strain *Lactobacillus paracasei* IMPC2.1 in an industrial plant. Int. J. Food Microbiol..

[47] AOAC (1990). Official Methods of Analysis.

[48] Bradford M.M. (1976). A rapid and sensitive method for the quantitation of microgram quantities of protein utilizing the principle of protein-dye binding. Anal. Biochem..

[49] Dubois M., Gilles K.A., Hamilton J.K., Rebers P.T., Smith F. (1956). Colorimetric method for determination of sugars and related substances. Anal. Chem..

[50] Osborne T.B. (1907). The Proteins of the Wheat Kernel (Monograph).

[51] Weiss W., Vogelmeier C., Gorg A. (1993). Electrophoretic characterization of wheat grain allergens from different cultivars involved in bakers’ asthma. Electrophoresis.

[52] Doi E., Shibata D., Matoba T. (1981). Modified colorimetric ninhydrin methods for peptidase assay. Anal Biochem..

[53] AOAC (2000). Official Methods of Analysis.

[54] American Association of Cereal Chemists (AACC) (2000). Approved Methods of the AACC.

[55] Lavermicocca P., Valerio F., Evidente A., Lazzaroni S., Corsetti A., Gobbetti M. (2000). Purification and characterization of novel antifungal compounds from the sourdough *Lactobacillus plantarum* strain 21B. Appl. Environ. Microbiol..

[56] Montemurro M., Pontonio E., Gobbetti M., Rizzello C.G. (2019). Investigation of the nutritional, functional and technological effects of the sourdough fermentation of sprouted flours. Int. J. Food Microbiol..

[57] Pétel C., Onno B., Prost C. (2017). Sourdough volatile compounds and their contribution to bread: A review. Trends Food Sci. Technol..

[58] Dantas T.L., Alonso Buriti F.C., Florentino E.R. (2021). Okra (*Abelmoschus esculentus* L.) as a potential functional food source of mucilage and bioactive compounds with technological applications and health benefits. Plants.

[59] Bi Q., Hong T., Mei X., Xu X., Xu D. (2023). Effect of high-molecular weight dextran-enriched sourdough fermented using *Leuconostoc mesenteroides* ATCC 8293 on bread quality and gluten. Food Biosci..

[60] Zhao C.J., Kinner M., Wismer W., Gänzle M.G. (2015). Effect of glutamate accumulation during sourdough fermentation with *Lactobacillus reuteri* on the taste of bread and sodium-reduced bread. Cereal Chem..

[61] Vermeulen N., Gänzle M.G., Vogel R.F. (2007). Glutamine deamidation by cereal-associated lactic acid bacteria. J. Appl. Microbiol..

[62] Peressini D., Sensidoni A. (2009). Effect of soluble dietary fibre addition on rheological and breadmaking properties of wheat doughs. J. Cereal Sci..

[63] Foschia M., Peressini D., Sensidoni A., Brennan C.S. (2013). The effects of dietary fibre addition on the quality of common cereal products. J. Cereal Sci..

[64] Ameh M.O., Gernah D.I., Igbabul B.D. (2013). Physico-chemical and sensory evaluation of wheat bread supplemented with stabilized undefatted rice bran. Food Nutr. Sci..

[65] Chareonthaikij P., Uan-On T., Prinyawiwatkul W. (2016). Effects of pineapple pomace fibre on physicochemical properties of composite flour and dough, and consumer acceptance of fibre-enriched wheat bread. Int. J. Food Sci. Technol..

[66] Plazzotta S., Sillani S., Manzocco L. (2018). Exploitation of lettuce waste flour to increase bread functionality: Effect on physical, nutritional, sensory properties and on consumer response. Int. J. Food Sci. Technol..

[67] Nawab A., Alam F., Haq M.A., Hasnain A. (2016). Effect of guar and xanthan gums on functional properties of mango (*Mangifera indica*) kernel starch. Int. J. Biol. Macromol..

[68] Di Cagno R., De Angelis M., Limitone A., Minervini F., Carnevali P., Corsetti A., Gänzle M., Ciati R., Gobbetti M. (2006). Glucan and fructan production by sourdough *Weissella cibaria* and *Lactobacillus plantarum*. J. Agric. Food Chem..

[69] Dong Y., Ronholm J., Fliss I., Karboune S. (2024). Screening of lactic acid bacteria strains for potential sourdough and bread applications: Enzyme expression and exopolysaccharide production. Probiotics Antimicrob. Proteins.

[70] Zhang Y., Wang D., Zhang Z., Guan H., Zhang Y., Xu D., Xu X., Li D. (2024). Improvement on wheat bread quality by in situ produced dextran—A comprehensive review from the viewpoint of starch and gluten. Compr. Rev. Food Sci. Food Saf..

[71] Nawrocka A., Krekora M., Niewiadomski Z., Miś A. (2018). Characteristics of the chemical processes induced by celluloses in the model and gluten dough studied with application of FTIR spectroscopy. Food Hydrocoll..

[72] Pepe O., Ventorino V., Cavella S., Fagnano M., Brugno R. (2013). Prebiotic content of bread prepared with flour from immature wheat grain and selected dextran-producing lactic acid bacteria. Appl. Environ. Microbiol..

[73] Sami R., Lianzhou J., Yang L., Ma Y., Jing J. (2013). Evaluation of fatty acid and amino acid compositions in okra (*Abelmoschus esculentus*) grown in different geographical locations. Biomed Res. Int..

